# Tissue specific considerations in implementing high intensity focussed ultrasound under magnetic resonance imaging guidance

**DOI:** 10.3389/fonc.2022.1037959

**Published:** 2022-11-01

**Authors:** Nandita M. deSouza, Wladyslaw Gedroyc, Ian Rivens, Gail ter Haar

**Affiliations:** ^1^ Division of Radiotherapy and Imaging, The Institute of Cancer Research, London, United Kingdom; ^2^ Faculty of Medicine, St. Mary’s Hospital, Imperial College, London, United Kingdom

**Keywords:** high intensity focussed ultrasound, soft tissue, fat, bone, vascular, magnetic resonance imaging

## Abstract

High-intensity focused ultrasound can ablate a target permanently, leaving tissues through which it passes thermally unaffected. When delivered under magnetic resonance (MR) imaging guidance, the change in tissue relaxivity on heating is used to monitor the temperatures achieved. Different tissue types in the pre-focal beam path result in energy loss defined by their individual attenuation coefficients. Furthermore, at interfaces with different acoustic impedances the beam will be both reflected and refracted, changing the position of the focus. For complex interfaces this effect is exacerbated. Moreover, blood vessels proximal to the focal region can dissipate heat, altering the expected region of damage. In the target volume, the temperature distribution depends on the thermal conductivity (or diffusivity) of the tissue and its heat capacity. These are different for vascular tissues, water and fat containing tissues and bone. Therefore, documenting the characteristics of the pre-focal and target tissues is critical for effective delivery of HIFU. MR imaging provides excellent anatomic detail and characterization of soft tissue components. It is an ideal modality for real-time planning and monitoring of HIFU ablation, and provides non-invasive temperature maps. Clinical applications involve soft-tissue (abdomino-pelvic applications) or bone (brain applications) pre-focally and at the target (soft-tissue tumors and bone metastases respectively). This article addresses the technical difficulties of delivering HIFU effectively when vascular tissues, densely cellular tissues, fat or bone are traversed pre-focally, and the clinical applications that target these tissues. The strengths and limitations of MR techniques used for monitoring ablation in these tissues are also discussed.

## Introduction

High Intensity focused ultrasound (HIFU), a precise thermoablative technique, has been utilized in the clinic for several decades. Although ultrasound guidance was initially preferred, the exquisite soft tissue contrast of magnetic resonance imaging (MRI) has driven MRI guidance and monitoring. Re-engineering of HIFU systems and MRI equipment has been necessary to accommodate these advances, which involve treatment of a variety of tissue types in the brain and body.

MRI delineates soft tissues by their widely different longitudinal and transverse relaxation properties. Adapting the radiofrequency (rf) pulse sequence means that tissue components such as densely cellular, vascular, cystic, fat containing or bone can be easily distinguished. Utilization of proton density and relaxation properties also enables derivation of temperature measurements in real-time, a technique referred to as MR thermometry. In the pre-focal region, the attenuation of the HIFU beam is determined by the tissues encountered by the beam (soft tissue, fat, bone), and any reflection or refraction of the beam affects the location of the focus. Heat dissipation through flowing blood in neighboring vessels also occurs. At the target, tissue characteristics also determine the temperature distribution of the tissue, for instance densely cellular, poorly vascular tissues may have high acoustic absorption coefficients, and disseminate heat less readily thus achieving higher temperatures. As MRI enables direct visualization of tissue characteristics both in the pre-focal region and at the target during and after HIFU ablation and can relate changes in tissue relaxation to temperature, this article provides a perspective on the implications of different tissue types in the pre-focal region and at the target when delivering HIFU under MR guidance (MRgHIFU).

## Vascular and non-vascular soft tissues

### Technical implications

In soft tissues, where hydrogen bonds are present, the decrease in the proton resonant frequency on heating (0.01 parts per million (ppm) per °C), results in a phase change measurable on a gradient-echo sequence. This proton resonant frequency shift (PRFS) in aqueous tissues changes linearly with temperature over a useful clinical range (-15°C to 100°C). It requires a reference measurement against which changes at the target are recorded. Data is acquired in real time concurrent with treatment delivery, and subtractions of the pre from post heating data allow temperature maps to be generated that are superimposed on the anatomic images for real time visualization of heating. Placement of an acquisition slice in a reference tissue requires a suitable soft tissue in the neighboring pre-focal region. Temperatures of 56°C for one second usually suffice, but higher temperatures are aimed for where a lot of blood vessels are present.

Air in the lungs precludes the use of HIFU for lung lesions. While lung flooding to provide acoustic coupling has been proposed, this is not yet used clinically (1). The use of HIFU in the thorax is therefore limited to treating chest wall lesions (2) and cardiac lesions (3–5), although the latter requires specialist catheter-mounted HIFU transducers. Extracorporeal delivery of HIFU to abdominal tumors through intercostal spaces has been achieved using dedicated phased arrays (6, 7) where careful design and phasing of the individual element arrays is necessary to avoid rib heating and focal splitting caused by interference effects (8).

### Clinical applications

One of the commonest applications of MRgHIFU is in the treatment of uterine fibroids. As fibroids are compositionally heterogenous with varying amounts of fibromuscular stroma, cysts, and calcification, the efficacy of the treatment has been highly variable with some fibroids responding better in terms of volume reduction and symptom control than others (9). The Funaki classification based on T2-weighted intensity compared to myometrium indicated that high intensity fibroids responded poorly and should not be considered for MRgHIFU. More recently this has been refined to use of T2 relaxation values and apparent diffusion coefficients (ADC) of the fibroid at baseline to predict outcome. It was initially assumed that poorer outcome was due to vascularity in T2 hyperintense fibroids (10) but more recently this phenomenon has been linked to increased amounts of extracellular water: shorter T2 and lower ADC values and thus more densely cellular fibroids have a better outcome (11, 12). This is echoed by data from a registry study in radio-recurrent prostate cancer, where the extracellular volume fraction in a multivariate analysis was shown to be the only independent predictor of poor progression-free survival (13). In brain tissue, where extracellular volume fraction differs between grey and white matter, thermal responses between them have been shown to differ in *ex vivo* data: the attenuation coefficient curves of white matter show a definite linear behavior in relation to temperature (14).

Conventional percutaneous ablation procedures such as radiofrequency and microwave ablation are compromised by heat sink from vessels close to the targeted point of heating (15). In contrast, focused ultrasound heats target tissues almost instantaneously with faster effects (16). Therefore, in comparison to percutaneous ablative techniques, treatment of deep tissues like the pancreas may not be as affected by the presence of vasculature close to the target. *In vivo* preclinical and clinical data supports this: HIFU lesions produced in the liver in live pigs extended up to and beyond patent vessels without damage or occlusion of the in-between vessel (16) with similar responses in human pancreas (17). Focused ultrasound is therefore less affected than percutaneous ablation techniques by the presence of significant vasculature in the surrounding tissues.

A small group of patients who have slow flow venous malformations, often with eddying flow, have been treated with HIFU (18). Very high power HIFU delivered over a short period has been shown to be reasonably successful but proximity to the skin risks severe heating and burns.

### Imaging the immediate and late effects of ablation in soft-tissues

As fibroid tissue is very inhomogeneous, responses produced to heating with focused ultrasound are variable. MR thermometry is accurate to ±1°C and can provide almost real time maps of heat deposition in tissues during the procedure. This allows titration of the HIFU parameters against the visualized thermal response so achieving a personalized treatment for each patient. In brain, where the tissue is much more homogeneous factors such as skull curvature and thickness affect the size and position of the focus. MR thermal monitoring at this point is critical for making sure that temperature deposition is precisely at the target since millimeters either side may cause significant untoward clinical side effects.

Post procedure monitoring of soft tissue ablation (fibroid, pancreas, prostate) utilizes contrast enhanced MRI with high temporal resolution to display the non-perfused volume. Post ablation the area of destruction can be visualized by postcontrast T1-weighted imaging. In the brain, postprocedural imaging requires T2-weighted imaging, diffusion-weighted imaging (19) and susceptibility-weighted imaging to display the very small area of ablative destruction. In a swine model diffusion-weighted and T1-weighted MR were used immediately post-procedure to depict ablation volume while T2 and fluid-attenuated inversion-recovery (FLAIR) images provided more accurate reflections of ablation volume after one week (20). It is common for the lesion to be barely visible at six months post procedure. Postcontrast sequences are less useful in these millimetre-sized brain lesions in comparison to conventional fibroid ablation where the volume of destruction is huge.

## Fat

### Technical implications

Where there is a lack of hydrogen bonds disruptable by heating, such as in pre-focal fat, it is not possible to generate MR thermometry measurements. All applications of HIFU in the body, such as the treatment of fibroids (9), pancreatic tumors (21) and pelvic tumors (22) involve traversing subcutaneous and abdominal fat of variable thickness, which may be layered with muscle of soft-tissue density. Because of the higher attenuation coefficient of fatty tissues compared to non-fatty soft tissues, e.g. muscle (23) treating lesions deep to fat such as renal tumors has proved difficult (24, 25). Increased absorption causes fat to heat more, and temperature induced differences in acoustic impedance between fatty and non-fatty soft tissues cause reflection and refraction of ultrasound energy at tissue interfaces, further influencing the degree and location of heating at the target. The limitations of delivering ablative doses to targets with varying distributions of pre-focal fat and muscle tissues have been demonstrated in an experimental model: focal thermal dose volumes were difficult to achieve at 8 cm depth with 6 cm pre-focal fat, even with 300W, ≥6kJ exposures (26). Using the known attenuation coefficients of the tissue mimics used, this work predicted that following a 300 W exposure less than 100 W would remain after passing through ≥4 cm of fat mimic compared to 120 W at 8 cm depth when muscle mimic was the only pre-focal tissue. There was also pre-focal heating seen in the fat mimicking model, but not in the muscle-only model (26), probably related to increased absorption (27). Thus, pre-focal fat not only limits thermal ablation for deep-seated lesions, but also increases the risk of pre-focal tissue damage. Layered distributions of fat/muscle/fat also cause the immediate pre-focal region to heat more than in fat and muscle only models (26). The increased energy transfer through the lower attenuating muscle layer coupled with the relatively high absorption in the fat mimic is the likely contributory factor. Reported attenuation coefficients of porcine adipose tissue have ranged from 0.8 ± 0.1 dB/cm at 1 MHz (28) to 2.7dB/cm at 1.1 MHz at 37°C (29), and of human perinephric fat from 0.8dB/cm (30) to 1.35dB/cm, when removed from the body, and held at room temperature. The measured acoustic characteristics of fat are therefore unlikely to be very accurate. As *in vivo* human tissues are more heterogenous than the layers of tissue mimic, there is an anticipated increased potential for reflection and refraction leading to greater energy losses *in vivo* especially when these layers are wedge shaped. Displacement of the focus caused by the temperature-dependent speed of sound demonstrated in porcine fat (24) also should be considered when positioning the focus at the treatment site.

Use of MRI relaxation times of fat are an alternative method of assessing temperature as they show temperature dependence (T_1_ changes by 1–2% per °C  (31) and T_2_ by ~3% per °C  (32). T2 measurements with water suppression have been purported to be more accurate (33), but they also yield higher values which require longer echo-trains for their measurement and thus longer acquisition times. In fatty breast tissues, the effect of heating has been shown to cause magnetic field disturbances with errors of up to 3.8°C that require correction with model-based approaches (34) Techniques such as high-bandwidth, multi-echo readouts added to a hybrid proton resonance frequency shift (PRFS)/T1 sequence also have been shown to improve the precision of temperature measurements (35). Unfortunately, the length of image acquisition for T2 relaxation means that significant cooling may occur when these sequences are utilized as part of monitoring HIFU treatments (32) and these cooling effects must be accounted for.

### Clinical applications

Subcutaneous fat thickness is recognized as a key factor in the successful ablation of uterine fibroids (36). Treating abdominal targets with fat in the pre-focal path has therefore used the prone position with water-filled balloons to compress the subcutaneous fat and also to displace any overlying bowel (21, 37). Approaches using bladder and rectal filling followed by bladder emptying have been successfully used to displace bowel (38), but have no impact on overlying subcutaneous fat. With deep-seated gynecological tumors, pre-focal gluteal fat has often resulted in inadequate tumor doses at the maximal available power of clinical systems (Sonalleve 300 W for 40 s using an 8 mm diameter cell). Higher acoustic power settings (>700 W) are possible but not currently enabled for clinical HIFU treatments for improving focal heating, without causing concurrent pre-focal damage. Other, differently configured extra-corporeal HIFU devices may improve dose delivery such as recently available longer focal length transducers and actively coupled cooling systems employed to achieve more effective target heating without the risk of skin burn. Intra-cavitary HIFU devices for tumors in the vicinity of the vagina, bladder or rectum (39) would also be an advantage, circumventing the problem of subcutaneous and gluteal fat.

### Imaging the effects of ablation in fatty tissues

HIFU has recently been advocated for the destruction of adipose tissue in cosmetic procedures to achieve fat reduction (40). In these circumstances the direct effects on the fat itself have not been assessed with imaging. In a preclinical porcine model, reduction in the thickness of the subcutaneous fat was demonstrated on ultrasound 90 days post treatment (41). MR visceral fat volumetric measurements have been used to demonstrate its reduction in volume after treatment with MRgHIFU in obese rats (33). This study also used T2 mapping to demonstrate a measurable increase in T2 relaxivity in the treated fat compared to the remote fat that occurred immediately and was sustained at 60 mins. Clinical studies have been limited to fat thickness measurements on ultrasound (42, 43). Imaging the thermal injury and post-treatment scar within fatty tissues *in vivo* has not been documented.

## Bone

### Technical implications for transcranial HIFU and bone targets

Both the density and the speed of sound are considerably higher in bone than in soft tissue, which results in an acoustic impedance mismatch and reflection of ultrasound energy at bone/soft tissue interfaces. Absorption and scatter of ultrasound also are higher in bone than in soft tissues (44). The absorption mechanisms that lead to a loss of energy within the focal region may be reduced slightly by using lower (than 1 MHz) ultrasound frequencies.

As the skull bone is neither flat nor uniformly thick, focal disruption can be compensated for by mapping the skull density and thickness spatially and using many element transducers to account for these variations so that a focal region will still form, in a technique known as time reversal. Patients with much more absorbent skulls with thicker volumes of marrow in the skull diploe greatly limit the amount of energy that can be deposited at the tissue site with most of the focused ultrasound energy being deposited within the skull vault itself. To produce a consistent lesion at the target site therefore under these varying conditions of skull thickness requires accurate temperature monitoring at the target with subsequent careful titration of the ultrasound parameters to achieve a consistent ablative lesion at the target site with millimetre accuracy.

In the abdomen, the rib cage forms a significant barrier to acoustic energy transmission, and ultrasound exposure over it carries with it the risk of skin burn. In body applications, the presence of bone can make tissues lying behind inaccessible to treatment.

### Clinical applications

Transcranial HIFU with bone as a pre-focal tissue has been successfully utilized to treat essential tremor (45, 46) and brain tumors (47) in a limited feasibility setting. A pilot clinical trial NCT01473485 has yet to complete and report. HIFU has also been trialled for bone targets in osteoid osteoma and as a locally ablative technique for painful bone metastases. In paediatric populations, small series of patients with osteoid osteomas have experienced remarkable pain relief; total pain resolution and cessation of analgesics were achieved in 88% of patients with refractory lesions after 4 weeks (48). For metastatic bony lesions, an international randomized-controlled phase III trial of 197 patients (49) showed that 64% responded in the treatment arm, (23% with a complete pain response defined as NRS=0 without >25% increase in analgesic consumption) compared to 20% in the placebo arm, making it a viable alternative therapy in these patients with intractable bone pain. A few other small studies further endorse its use in this clinical setting (50–56). The mechanism of action by which HIFU palliates pain is not fully understood. Thermal denervation of the periosteum has been postulated (57) and so treatment strategies have targeted the bone surface (58) where acoustic absorption is high and thermal conduction low thus achieving high temperatures at the periosteum on the bone surface. However, if sufficient thermal energy is also transmitted through the cortex, there may be additional effects from tumor debulking (59) or from alterations in the release of pro-inflammatory signaling molecules (60). In a cohort of 21 patients, better pain response rates were seen in patients with intra-osseous lesions (67% for intraosseous lesions vs 33% for extraosseous lesions) (61), supporting the hypothesis that HIFU thermally denervates the periosteum. In the intra-osseous patients, thermal neurolysis was probably achieved, given clear regions of focal non-enhancement seen immediately after treatment at the bone surface in 55%, and by Day 30 in 78% of cases.

### Imaging the immediate and late effects of ablation in bone

Ablative heating at the periosteum cannot measure temperature in fatty bone marrow (62, 63), or at the cortex, so temperature is inferred by measuring temperature rise in adjacent aqueous soft tissues. In ex vivo tissues, baseline PRFS has been shown to be accurate to 1°C and to regain baseline temperatures within 5 min (64). T1-weighted imaging shows reduced contrast enhancement immediately after treatment (50); a non-perfused volume was recognized immediately after treatment in 8 of 9 patients with intra-osseous tumors that persisted at Day 60 and 90 (61). Extra-osseous bone metastases have imaging changes that are harder to define and quantify as the appearances on baseline scans are usually heterogenous. Where change is evident, there may be a definite increase in the non-perfused area or a reduction in contrast enhancement in some cases, while in others ill-defined expansion of non-perfused regions has been identified (61). In *ex vivo* studies, the ADC in bony targets increases in the muscle adjacent to the sonicated periosteum and is maximal after 1-5 min, with a documented coefficient of variation for repeat ADC measurements of 0.8%. Changes that persist beyond 20 min appear to remain stable for 2 h and correlate significantly with thermal parameters. A 20% ADC increase was shown to result in macroscopic tissue damage (64). In a clinical setting the ADC in treated lesions was significantly higher after 1 month of treatment and remained so at 6 months follow-up (56). Ultrashort echo-time (UTE) MRI sequences capture signals from tissues such as bone which have a very short transverse relaxation time, have only really been trialled in transcranial applications to image the skull and reduce the need for computerized tomography (CT) guidance. Using UTE-based skull intensity information, Guo et al. demonstrated the possibility of replacing CT guidance for transcranial MRgHIFU because of the equivalence of thermal profiles and focal locations (65). The use of UTE-MRI for monitoring the appearance and evolution of bone ablation remains to be explored.

## Summary and conclusions

**Figure 1:**
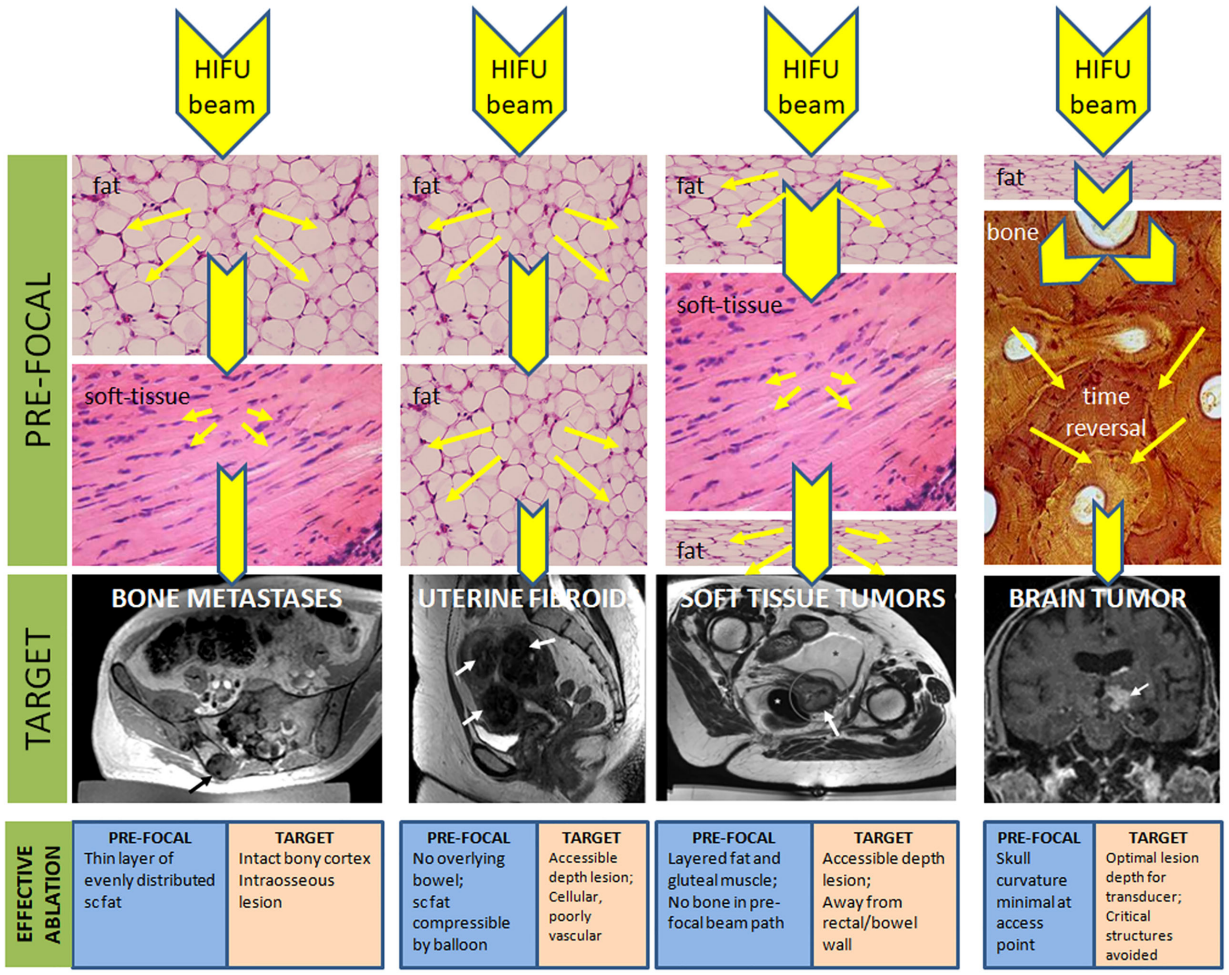
Pre-focal and target tissue considerations for achieving effective thermal ablation with HIFU. Reduction in the beam energy as it traverses tissues with different characteristics in the pre-focal region are given by reduction in size of the yellow arrowheads (not to scale) because of dispersion of the beam energy (yellow arrows). With bone in the pre-focal path, a time reversal technique is required to re-focus the beam. Favorable considerations of the pre-focal and target tissues to achieve effective ablation are indicated.

Tissue characteristics profoundly influence the delivery, monitoring and follow-up of HIFU (**Table 1**). MRgHIFU enables critically important thermometry information, but T1-W PRFS methods do not work when fat is the predominant pre-focal tissue. Reflection and refraction of ultrasound at bone-soft tissue interfaces need compensation in transcranial applications but may prove advantageous in treating painful intraosseous bone metastases. The ability to characterize tissue together with anatomical information makes MRI the ideal imaging modality as it offers options for modification and optimization of treatment based on the characteristics of pre-focal and target tissues (**Figure 1**).

**Table 1:** Categories, clinical need and tissue characteristics of lesions treated with HIFU.

Category	Clinical Application	Intended outcome	Target tissue characteristic	Pre-focal tissue characteristic	Energy/Power levels used	Considerations
**Abdomino-pelvic tumors**	Uterine fibroids	Relief of menstrual pain/bleeding	Soft tissue	Mainly fat, muscle layer (Overlying bowel)	Up to 200W, 4-16mm diameter treatment cells (66, 67)	• Long T2 fibroids, vascular fibroids difficult to treat * Deep lesion with thick layer of subcutaneous fat difficult to treat • Overlying bowel not displaceable
	Pancreatic tumors	Debulking for neoadjuvant therapy, part of multimodality treatment, pain relief in palliative setting	Soft tissue	Mainly subcutaneous and visceral fat, muscle layer (Overlying bowel)	Total applied energy 344 ± 152kJ, power 200-400W (68)	* Deep seated tumors with overlying bowel difficult to treat
	Recurrent gynecological tumors	Pain relief in palliative setting	Soft tissue	Mainly fat, muscle layer	Total applied energy 57 ± 29kJ, power up to 300W, 4-8mm treatment cells (22)	• Deep seated tumors with thick layer of overlying fat inaccessible by current systems • Uneven fat and muscle in prefocal region distorts focus • Pelvic bone in the beam path blocks beam
	Prostate cancer	Tumor ablation for early disease	Soft tissue	Rectal wall in transrectal approach	Variable depending on transrectal, transurethral or extracorporeal approach	• Mostly endorectal approach • Limited availability of MR guided systems
	Other (sarcoma, renal, liver)	Tumor debulking	Soft tissue	Subcutaneous/visceral/peri nephric fat	Power 5-10kW/cm^2^ (69); power 5-15kW/cm^2^ (140-260 W) (70)	* Beam attenuation by perinephric/visceral fat
**Brain**	Basal ganglia/Thalamus	Resolution of essential tremor	Soft tissue	Bone	Mean highest energy 22.8 ± 8.5kJ (45); maximum energy 12kJ per sonication (71)	• High degree of lesioning accuracy is critical
	Brain tumors	Tumor debulking	Soft tissue	Bone	12kJ per sonication (1200W for 10s or 800W for 20s) (72)	• Requires intraoperative procedure and stereotactic frame
**Bone**	Osteoid osteomas	Pain relief	Bone	Overlying skin	Total applied energy 1180 ± 736 J (73)	• Pediatric population • Potential for skin burns
	Metastatic bone tumors	Pain relief in palliative setting	Bone and soft tissue	Skin, subcutaneous fat	Intraosseous: power 70 ± 30W per sonication, Total applied energy 24 ± 17kJ; extraosseous: power 85 ± 47W per sonication, Total applied energy 52 ± 49 kJ (61)	• Ineffective if large extraosseous component • Treatment of vertebral lesions limited by need to traverse thecal sac in prefocal region
**Breast**	Fibroadenomas, breast cancer	Lesion ablation	Soft tissue	Breast fat, glandular breast tissue	Total applied energy 134.6 ± 19.3J, power 33.3 ± 4.8W (74); 5-15 kW/cm^2^ (75)	• Not cost-effective over surgery, cosmetic advantages are minor
**Subcutaneous Fat**	Lipolysis	Cosmetic	Fat	Overlying skin	Power 72W, energy density up to 206J/cm^3^ at skin surface (76)	• Competing technologies for cosmetic fat reduction • Ultrasound guided, no MR thermometry

The energies used and key considerations hindering wider clinical adoption are detailed.

## Data availability statement

Any original contributions presented in the study are included in the article/supplementary material. Further inquiries can be directed to the corresponding author.

## Author contributions

All authors contributed to the planning, drafting, writing and editing of this Perspectives article. All authors contributed to the article and approved the submitted version.

## Conflict of interest

The authors declare that the research was conducted in the absence of any commercial or financial relationships that could be construed as a potential conflict of interest.

## Publisher’s note

All claims expressed in this article are solely those of the authors and do not necessarily represent those of their affiliated organizations, or those of the publisher, the editors and the reviewers. Any product that may be evaluated in this article, or claim that may be made by its manufacturer, is not guaranteed or endorsed by the publisher.
